# Transoral Cordectomy with Microelectrodes (TOMES) on an Outpatient Basis: Advancing Patient Comfort and Personalized Care Through Predictive Models

**DOI:** 10.3390/jpm15100465

**Published:** 2025-10-01

**Authors:** Cristina Rodríguez-Prado, Natsuki Oishi, Ernesto Fernández-Vidal, José Ramón Alba-García, Enrique Zapater

**Affiliations:** 1 ENT Department, Valencia University General Hospital, 46014 Valencia, Spain; rodriguez_cripra@gva.es (C.R.-P.); ernestofernandez@bellvitgehospital.cat (E.F.-V.); alba_josgar@gva.es (J.R.A.-G.); enrique.zapater@uv.es (E.Z.); 2 Faculty of Medicine and Odontology, Valencia University, 46010 Valencia, Spain

**Keywords:** cordectomy, laryngeal cancer, microelectrode, quality of life, healthcare costs, individual predictive model

## Abstract

**Background/Objectives**: Outpatient surgery enhances patient comfort while reduces surgical wait times and healthcare costs compared to inpatient procedures. This study evaluates the individual feasibility of performing transoral trans muscular cordectomies with microelectrodes (TOMES) on an outpatient basis. **Methods**: This observational study analyses TOMES types III, IV, and V cordectomies performed from January 2002 to December 2023. Key metrics include patient demographics, procedural details, incidence of bleeding, anticoagulation and other comorbidities. **Results**: Of the 143 procedures, 127 were cancer-related, while 16 were due to bilateral vocal cord paralysis. The average age was 65, with a predominantly male cohort (92%). Postoperative hemorrhage occurred in four cases, primarily among oncological patients, but there was no correlation with anticoagulation therapy. A personalized predictive model for bleeding risk was developed based on patient-specific characteristics and observed outcomes. Additionally, performing the procedure on an outpatient basis decreased healthcare costs and wait times for patients with T1/T2 glottic carcinoma. **Conclusions**: The findings indicate that TOMES type III or higher cordectomies can be safely performed on an outpatient basis, through the use of a personalized predictive model for each case and with appropriate postoperative monitoring. This approach has the potential to lower healthcare costs and improve patient quality of life through individualized assessments and structured risk analysis.

## 1. Introduction

Laryngeal cancer represents approximately one-third of all head and neck malignancies, with a reported 5-year overall survival rate ranging from 50% to 60% [1]. It remains a significant cause of morbidity and mortality worldwide. Among laryngeal tumours, the glottic region is the most commonly affected site, accounting for nearly half of the cases, although this proportion may vary depending on the population and geographical region studied [2]. The widespread use of advanced diagnostic tools such as high-definition videolaryngoscopy and narrow-band imaging has facilitated the earlier detection of glottic carcinomas. Consequently, an increasing number of cases are now diagnosed at early clinical stages (T1–T2), allowing for less invasive and organ-preserving treatment approaches [3]. For early stage glottic cancer, both transoral surgery and radiotherapy are considered standard treatment options. While some studies suggest that surgical intervention may offer better local control, faster recovery, and lower treatment-associated morbidity, other analyses report no statistically significant differences in oncologic outcomes between the two modalities [3,4]. However, surgery has been shown in some contexts to offer a more favourable cost-effectiveness profile compared to radiotherapy [5].

Among the surgical approaches, transoral microelectrode surgery (TOMES) has emerged as a reliable alternative to laser microlaryngoscopy. TOMES enables precise dissection with simultaneous coagulation, preserves tactile feedback, reduces operative time, and is significantly more cost-efficient than CO_2_ laser systems [6,7].

**Figure 1:**
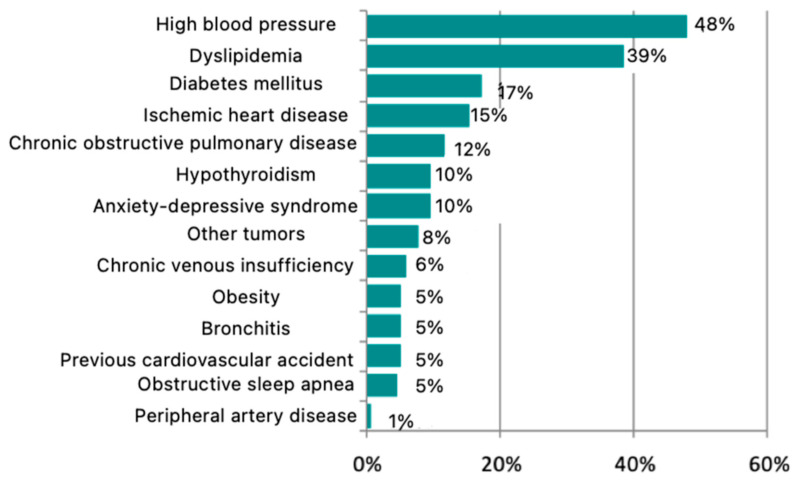
Concomitant diseases of the patients under study.

In these cases of T1/T2 glottic tumours, the indicated surgical procedure is the cordectomy, which consists of the partial or complete excision of the vocal fold, typically performed for oncological or functional indications. The extent and depth of resection is classified according to the system proposed by the European Laryngological Society (ELS) [8,9], which provides a standardized framework for endoscopic cordectomy procedures. This classification is based on anatomical criteria and distinguishes between six main types (Figure 1):

Type I: Subepithelial cordectomy

Type II: Subligamental cordectomy

Type III: Transmuscular cordectomy

Type IV: Total cordectomy

Type V: Extended cordectomy

Type VI: Cordectomy involving the anterior commissure

Furthermore, type V cordectomies are subdivided depending on the anatomical structures involved:

Va: Extension to the contralateral vocal fold including the anterior commissure

Vb: Involvement of the arytenoid

Vc: Extension into the subglottic space

Vd: Involvement of the laryngeal ventricle

This classification allows surgeons to plan the extent of resection with greater precision based on tumour size, location, and infiltration depth, and it facilitates comparison of surgical outcomes across centres. Type III, IV, and V cordectomies entail deeper tissue resection and carry a higher theoretical risk of postoperative complications such as bleeding or prolonged hospitalization. Assessing these more complex procedures is crucial to determine the safety and feasibility of implementing outpatient protocols for selected patients.

In our institution, TOMES has been the standard technique for performing cordectomies for over two decades, consistently yielding excellent oncological outcomes with minimal postoperative complications. This experience has fostered the expansion of TOMES procedures into the outpatient setting.

In parallel, there has been a growing global trend toward ambulatory surgery across multiple specialties. In addition, medicine is increasingly being practised in a more personalized way, with each case being assessed individually and treatment always adapted to the situation and condition of each patient at any given time.

Ambulatory surgical models tailored to each person offer numerous benefits, including enhanced patient satisfaction, reduced hospital-acquired complications, and faster return to daily activities [10]. From a health systems perspective, same-day discharge protocols reduce healthcare expenditures and optimize surgical wait times by improving the turnover of operating room resources [11,12].

In this context, assessing the safety and individual feasibility of performing more extensive cordectomies for glottic tumours in an outpatient setting is of growing clinical relevance. Understanding the predictors of postoperative complications, particularly bleeding and prolonged hospitalization, is essential to refine patient selection criteria and ensure optimal perioperative outcomes.

The primary objective of this study is to analyze the individual risk factors associated with postoperative complications, particularly bleeding and prolonged hospital stay, in patients undergoing transmuscular or extended cordectomies (types III, IV, and V) using transoral microelectrode surgery (TOMES).

Based on this analysis, we aim to develop personalized predictive models that can estimate the likelihood of successful outpatient management for each patient. The ultimate goal is to support clinical decision-making regarding ambulatory surgery eligibility, with the intention of enhancing patient comfort and quality of life, optimizing hospital resource allocation, reducing surgical waiting times, and lowering overall healthcare costs.

## 2. Materials and Methods

A retrospective observational study was conducted at a single tertiary care centre, based on the review of medical records from patients who underwent transoral microelectrode surgery (TOMES) for transmuscular (type III) or extended (types IV and V) cordectomies between January 2002 and December 2023 [8,9].

Eligible patients were those who underwent type III, IV, or V cordectomies using TOMES and fulfilled all protocol-defined criteria. Exclusion criteria included intraoperative conversion to open surgery, the use of alternative surgical instrumentation, or any deviation from standard surgical indications. Furthermore, type I and II cordectomies were excluded, as these are currently performed on an outpatient basis at our institution.

Based on epidemiological data from the Spanish Network of Cancer Registries (REDECAN 2025), the overall incidence of laryngeal cancer in Spain is estimated at 6.1 cases per 100,000 inhabitants per year when both sexes are considered. Approximately 60% of laryngeal cancers originate in the glottis, and two-thirds of glottic carcinomas are diagnosed at an early stage. In our healthcare area, which serves a population of approximately 390,000 inhabitants, the expected incidence of early-stage (T1–T2) glottic squamous cell carcinoma was therefore estimated at 2.5 cases per 100,000 person-years, corresponding to 9–10 new cases annually.

Assuming a Poisson distribution for rare events, the required sample size was calculated to achieve a 95% confidence interval with a predefined relative precision. The number of cases was derived using the formula: *k* ≈ (*r* × 1.96)^2^ where *k* represents the required number of cases and *r* the desired relative margin of error.

To estimate the incidence with a relative precision of ±20%, a minimum of approximately 96 cases was required. Based on the expected annual incidence within our reference population, the accrual period was planned accordingly to meet this requirement.

Out of 151 cordectomies initially performed with TOMES, 8 cases were excluded due to conversion to open surgery. Thus, a total of 143 cordectomies met the inclusion criteria and were included in the final analysis.

Baseline variables included sex, age, underlying pathology (glottic carcinoma or vocal cord paralysis), and risk factors such as smoking and alcohol use. Comorbidities were recorded, including arterial hypertension, dyslipidemia, ischemic heart disease, diabetes mellitus, chronic obstructive pulmonary disease (COPD), hypothyroidism, anxiety-depressive disorder, malignancy at another site, chronic venous insufficiency, obesity, chronic bronchitis, prior cerebrovascular accident, obstructive sleep apnoea, and peripheral arterial disease. Surgical data encompassed the type of cordectomy and anticoagulant use on the day of surgery. Oral anticoagulants (including vitamin K antagonists, direct thrombin inhibitors, and factor Xa inhibitors) were discontinued 48 to 72 h before surgery, following hematology department recommendations. Bridging therapy with low-molecular-weight heparin (LMWH) was initiated and maintained until 24 to 48 h after the procedure, provided no adverse events occurred. Oral anticoagulation was then reintroduced according to clinical stability and standard protocol. Postoperative variables included the occurrence and timing of bleeding and the duration of hospital stay.

For qualitative variables, analytical tools (frequency tables) and graphical tools (pie charts and bar charts) were used. For quantitative variables we used analytical tools such as location statistics (means and medians), dispersion (standard deviations, percentiles and ranges) and shape statistics (symmetry and kurtosis coefficient), as well as graphical tools (histograms and box plots).

The relationship of postoperative bleeding with each of the baseline variables, concomitant diseases and surgical characteristics was analyzed.

First, statistical tests were applied to see the relationship of each variable with surgical bleeding, and then an estimation of the risk of bleeding was carried out for each of the characteristics considered. Finally, logistic regression models were constructed to adjust the probability of bleeding according to each of the factors considered in the study.

The relationship of hospitalization time with each of the baseline variables, concomitant diseases, surgical characteristics and the presence of bleeding after surgery was analyzed.

Firstly, statistical tests were applied to see the relationship of each variable with the length of hospitalization. In this group of patients, the need for long hospitalisations is very low, so it was decided to recode the days of hospitalization as a categorical variable (one day vs. more than one day).

For all statistical analyses, the R software version 4.4.1 has been used. In all cases, bilateral tests have been applied using a significance level of 5%, obtaining exact *p*-values whenever possible.

## 3. Results

A total of 143 surgical procedures meeting the inclusion criteria were performed. Of these, 127 were carried out in oncologic patients (122 with squamous cell carcinoma and 5 with verrucous carcinoma) while the remaining 16 cases corresponded to other indications, such as unilateral vocal cord paralysis. The cohort was predominantly male (92%, n = 131), with a mean age of 65 years. Regarding exposure to toxic habits, 108 patients (75.5%) reported at least one: 84 (58.7%) were active smokers, and 71 (49.7%) consumed alcohol. Notably, 47 patients (32.9%) presented with both risk factors (Table 1).

Concerning comorbidities, 19.6% of patients (n = 28) had no associated medical conditions at the time of surgery. However, 80.4% (n = 115) presented with at least one concomitant disease. The most common subgroup consisted of patients with two comorbidities, accounting for 25.9% of the total. The most prevalent comorbidity was arterial hypertension, affecting 48% of patients, followed by dyslipidemia (39%), diabetes mellitus (17%), ischemic heart disease (15%), chronic obstructive pulmonary disease (12%), hypothyroidism (10%), anxiety-depressive syndrome (10%), other malignancies (8%), chronic venous insufficiency (6%), obesity (5%), bronchitis (5%), previous cerebrovascular events (5%), obstructive sleep apnea (5%), and peripheral arterial disease (1%) (Figure 1).

**Figure 2:**
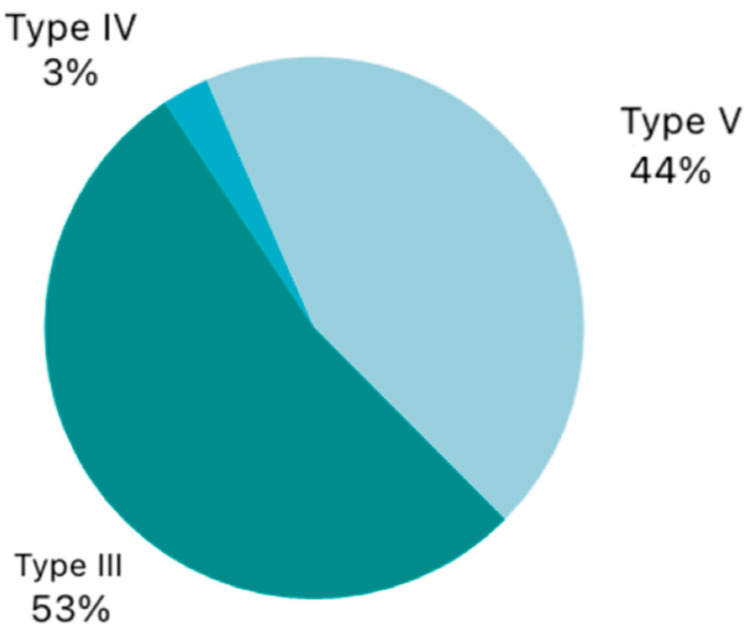
Types of cordectomies performed.

Regarding the type of surgical procedure performed, the majority of patients underwent a transmuscular cordectomy (Type III), accounting for 53.1% of cases. This was followed by extended cordectomy (Type V) in 44.1% of patients. A small subset underwent a complete cordectomy (Type IV), representing only 2.8% of the total (Figure 2).

Additionally, seven patients (4.9%) were under anticoagulant therapy on the day of surgery, none of whom experienced postoperative bleeding.

**Figure 3:**
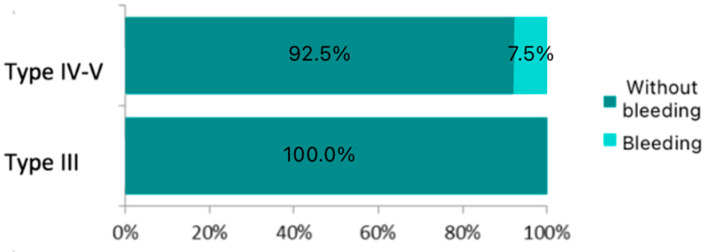
Percentage of bleeding by type of cordectomy.

A total of five postoperative bleeding events were recorded, corresponding to an estimated bleeding risk of 3.5% (95% CI: 1.5–7.9%) for this type of procedure performed with microelectrodes. The mean time to bleeding onset was 120 min after the end of surgery. The earliest case occurred immediately after extubation, while the latest occurred at 210 min postoperatively. No bleeding events were observed between 210 min and the seventh postoperative day, nor was any bleeding detected after the seventh postoperative day. Postoperative bleeding occurred in 8% of women and 3% of men. Four bleeding events were recorded in the oncologic group and one in the non-oncologic group. Approximately 5% of patients were under anticoagulant therapy; however, none of these patients experienced bleeding. Arterial hypertension was present in 49% of the total cohort, and 40% of the patients with bleeding had this condition. No statistically significant differences were found in postoperative bleeding by sex (*p* = 0.359), with an estimated risk of 3.1% in men (95% CI: 1.2–7.6%) and 8.3% in women (95% CI: 1.5–35.0%). Likewise, no significant difference was observed in median age between patients without bleeding (66 years) and those with bleeding (61 years) (*p*-value = 0.377). There was also no significant association between bleeding risk and underlying pathology (*p*-value = 0.554), with an estimated bleeding risk of 6.3% in patients with bilateral vocal cord paralysis and 3.3% in those with squamous cell carcinoma. No significant differences were observed based on smoking status (*p*-value = 0.649), with an estimated bleeding risk of 4.8% in smokers and 1.7% in non-smokers. Similarly, no significant differences were found with respect to alcohol consumption (*p*-value = 0.681), with an estimated bleeding risk of 4.2% in alcohol users and 2.8% in non-users. A statistically significant difference was observed in bleeding risk according to the type of cordectomy performed (*p*-value = 0.021), with no bleeding reported in patients who underwent a transmuscular cordectomy (Type III) and a 7.5% risk observed in those who underwent total or extended cordectomy (Type V). Patients undergoing Type IV–V cordectomies had an odds ratio of 13.464 for postoperative bleeding compared to those who underwent Type III procedures, a statistically significant result (*p*-value = 0.017). No other factors showed a statistically significant association in the univariate models, although bronchitis and sex demonstrated the highest levels of association among the remaining variables. These differences are illustrated in Figure 3.

Finally, the estimated bleeding risk was 0.0% in patients receiving anticoagulant therapy, compared to 3.7% in those not on such medication.

A multivariate model was constructed including those variables that showed a *p*-value < 0.25 in the univariate analysis. Table 2 summarizes the variables included in the univariate and multivariate analyses, indicating the level of statistical significance achieved with a 95% confidence interval.

**Figure 4:**
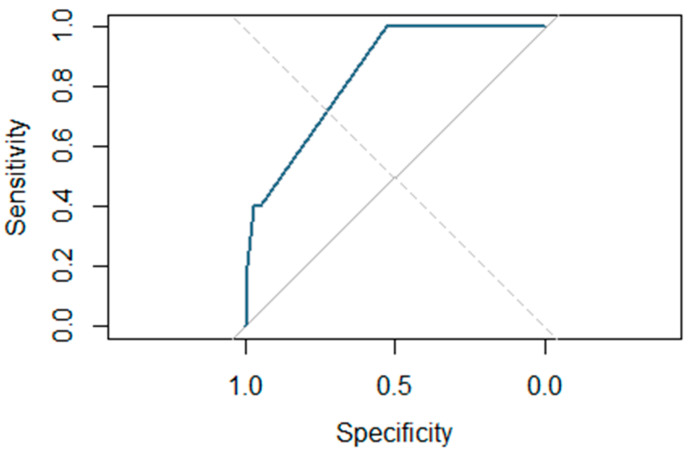
Predictive model for postoperative bleeding. The ROC curve associated with the final multivariate predictive model, which demonstrates high discriminatory power, with an area under the curve (AUC) of 0.839.

In the final model, the type of cordectomy emerged as the only statistically significant prognostic factor. However, factors such as gender (*p* = 0.056) and chronic bronchitis (*p* = 0.136) have also been included due to their proximity to statistical significance in the multivariate model, which increases its discriminatory power compared to the univariate model. All of this is reflected in Figure 4, which shows the ROC curve associated with the final multivariate predictive model, which demonstrates high discriminatory power, with an area under the curve (AUC) of 0.839—higher than that of the univariate model (AUC = 0.775).

**Figure 5:**
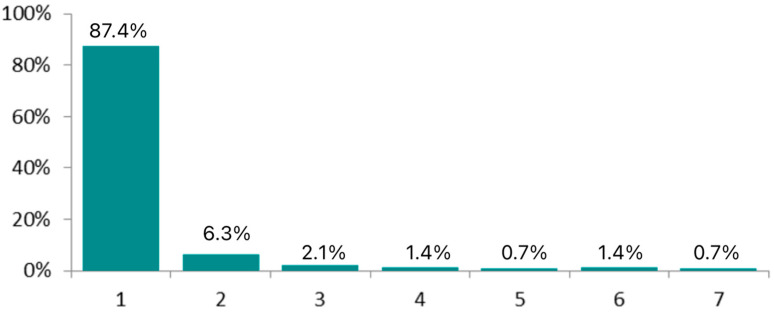
Days of hospitalization after surgery.

The mean hospital stay was 1.29 days, ranging from 1 to 7 days. Figure 5 illustrates the distribution of hospital stay duration in this patient cohort, showing that nearly all patients (87.4%) required only a single day of hospitalization.

Although no statistically significant differences were observed, a trend was noted suggesting a relationship between smoking status and length of hospital stay (*p*-value = 0.105), with smokers exhibiting a longer hospitalization period compared to non-smokers.

Statistically significant differences were found in the number of hospitalization days based on the presence or absence of anxiety-depressive syndrome (*p*-value = 0.032).

Patients with ischemic heart disease also showed a tendency toward longer hospital stays, although this was not statistically significant (*p*-value = 0.119).

The type of cordectomy performed approached statistical significance in relation to hospitalization duration (*p*-value = 0.137), with longer stays observed in patients who underwent total or extended cordectomy.

Finally, a highly significant difference was observed in hospitalization time between patients who experienced surgical bleeding and those who did not (*p*-value < 0.001).

When constructing the multivariable predictive model, postoperative bleeding was identified as a significant predictor of hospital stay longer than one day (*p*-value < 0.001).

**Figure 6:**
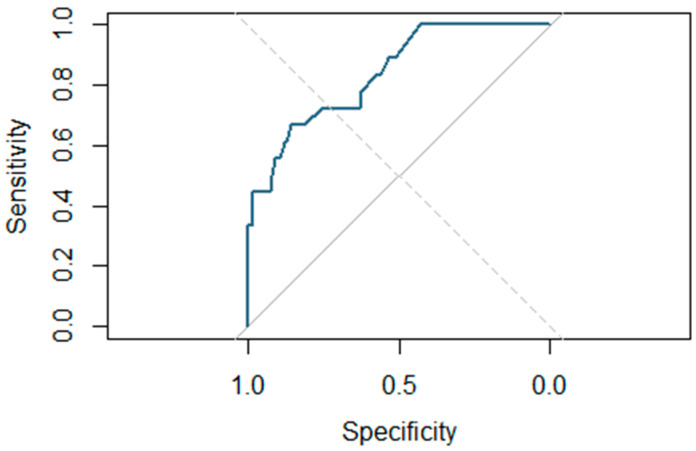
Predictive model for hospital stay. The ROC curve associated with the full multivariate predictive model including these variables, demonstrating good discriminative ability with an area under the curve (AUC) of 0.837.

For the construction of this final penalized multivariate logistic regression model, factors with a significance level lower than 0.25 in the univariate model were included (smoking *p* = 0.083; ischaemic heart disease *p* = 0.144; anxiety–depression syndrome *p* = 0.088 and type of pathology *p* = 0.112) were included in the model, given that the small number of events observed makes it difficult to adjust models with many independent variables. Smoking status and the presence of certain comorbidities approached statistical significance and may reach significance with an expanded sample size. Figure 6 shows the ROC curve associated with the full multivariate predictive model including these variables, demonstrating good discriminative ability with an area under the curve (AUC) of 0.837. This performance surpasses that of the model including only postoperative bleeding, which had an AUC of 0.639. The improvement of the full model over the bleeding-only model was statistically significant (*p*-value < 0.001).

Lastly, the costs incurred by the patients included in this study for the National Health System have been analyzed.

The total average cost per patient for outpatient cordectomy using TOMES was between EUR 963.82 and EUR 997.05, while procedures requiring hospitalization ranged from EUR 3523.15 to EUR 4651.67 (taking into account not only the surgery but also the stay in the post-anesthesia recovery unit, administrative costs, staff and materials). This represents an additional cost of between EUR 2559.33 and EUR 3688.85 when this surgery is performed on an inpatient basis, making inpatient surgery approximately 3.6 to 4.7 times more expensive than outpatient treatment. Assuming that hospitalization costs are the main factor explaining this difference, the excess cost corresponds to an estimated 2.3 to 4.1 days of post-surgical hospitalization (based on an average daily cost of between EUR 900 and EUR 1100). These results suggest that, although the surgical component itself is comparable, hospitalization significantly increases the total cost.

## 4. Discussion

The decision to perform a surgical procedure on an outpatient basis is inherently complex. It must take into account variables related to the underlying pathology, the surgical technique itself, the characteristics of the hospital, and, crucially, individual patient factors, both medical and social. These considerations guide the choice of the most appropriate and safest option for each patient, aiming to maximize well-being and potentially improve quality of life.

Before initiating outpatient surgical planning, it is essential to review the ambulatory surgery eligibility criteria specific to the institution. These typically include optimal control of chronic diseases, availability of adequate postoperative surveillance during the first 24 h, access to private transportation to the nearest hospital equipped to manage postoperative complications, and the patient’s proximity to that facility. Once these criteria are met, case-by-case evaluation allows us to offer safe outpatient surgery on an individual and personalized basis for each patient.

Over 80% of patients presented with at least one comorbidity, with arterial hypertension being the most common, present in nearly half of the cohort. This is a relevant finding considering the historical association between hypertension and increased risk of systemic bleeding [13]. However, in our analysis, the presence of hypertension did not correlate with a higher postoperative bleeding risk when cordectomies were performed using the TOMES technique. Combined with the low incidence of bleeding within the first 7 postoperative days, these results support the inclusion of well-controlled hypertensive patients in outpatient surgery protocols.

A similar trend was observed in anticoagulated patients. Although anticoagulant therapy, as well as coagulopathies or other haemostatic disorders, is classically associated with increased bleeding risk [14], our findings suggest that, with appropriate perioperative management, these patients did not experience a significantly higher incidence of bleeding when treated with the TOMES approach.

No bleeding events were observed beyond 210 min postoperatively. This suggests that there is no need to extend the usual postoperative recovery room monitoring time, which typically ranges from 6 to 8 h in most ambulatory surgical procedures. Furthermore, to ensure patient safety, no bleeding events were recorded between 210 min postoperatively and the 7th postoperative day in our sample. In other words, no haemorrhagic complications occurred at home after hospital discharge.

No statistically significant differences were observed in bleeding risk based on the underlying pathology (glottic carcinoma vs. vocal cord paralysis). Although bilateral vocal fold paralysis is not typically considered a risk factor for increased bleeding, in this study, a higher proportion of bleeding events was observed in these cases. This finding may be influenced by the relatively small number of patients with this pathology included in the cohort compared to those with tumoral lesions.

This finding warrants further investigation in future studies, as tumoral pathologies are generally associated with a higher bleeding tendency due to their rich vascularization, which often includes aberrant and fragile vessels [15].

While no significant differences were observed in bleeding risk by sex, smoking, chronic bronchitis, obesity, diabetes, dyslipidemia, etc., a statistically significant association was found between the type of cordectomy and postoperative bleeding. Specifically, extended cordectomies (Types IV and V), which involve resection beyond a single vocal fold [8,9], were associated with a 7.5% higher risk of bleeding. This finding is consistent with the expected increase in vascular injury in more extensive resections, especially when optimal haemostasis is not achieved. It is also possible that this association is indirectly related to tumour size, as larger tumours typically require more extensive surgical resections, which, as observed in this study, are associated with an increased risk of postoperative bleeding.

Other previously discussed variables, such as sex, chronic bronchitis, and smoking status, did not reach statistical significance; however, a trend toward significance was observed in relation to an increased risk of postoperative bleeding. These factors may potentially influence bleeding outcomes but the limited number of bleeding events and the overall low incidence of this complication in TOMES cordectomies may have reduced the power to detect significant differences.

For these reasons, developing and applying an individualized risk prediction model for postoperative bleeding is a valuable next step. Expanding the sample size in future studies will allow for a more refined analysis of variables that, while not statistically significant in the current cohort, have shown clinically relevant trends.

Although transoral laser cordectomies have been performed on an outpatient basis in some centres for years [16], cordectomies performed using transoral robotic surgery (TORS) have only recently been introduced, and indications for this surgical approach are steadily increasing. Furthermore, an expanding number of centres are incorporating this technique into their clinical practice [17].

Because of these reasons, this predictive risk model would not only be applicable when using microelectrodes as the surgical instrument but could also be extrapolated to optimize patient selection for transoral laser [16] or TORS cordectomies [17]. In doing so, it would contribute to optimizing outpatient surgical pathways and enhancing safety in procedures involving the upper airway.

Furthermore, we analyzed hospital length of stay in this cohort, aiming to identify which factors may contribute to prolonged hospitalization, particularly relevant given the known increase in hospitalization and elevated complications associated with extended inpatient stays. Postoperative bleeding was significantly associated with a longer hospital stay, as additional days of monitoring are required to ensure adequate haemostasis and optimal recovery. However, bleeding was not the only factor contributing to prolonged hospitalization. Other relevant risk factors were identified that should also be considered in the preoperative assessment. As discussed earlier, more extensive surgeries, which are often required for larger tumours, are associated with a higher risk of bleeding and, indirectly, with longer hospital stays due to the need for extended surveillance. Although not reaching statistical significance, a clear trend toward longer hospital stays was observed in smokers, patients with ischemic heart disease, and those with anxiety-depressive disorders. In the multivariable logistic regression model, the risk of prolonged hospitalization was notably higher in patients presenting with one or more of these four risk factors. These patient characteristics should therefore be incorporated into a predictive model for hospital length of stay.

On the other hand, transoral laser cordectomies have been associated with lower treatment costs for T1–T2 glottic tumours compared to open cordectomy or radiotherapy [5]. In addition, it is important to highlight the economic savings achieved by performing this surgery using transoral microelectrode surgery (TOMES) instead of laser, while still offering comparable clinical outcomes [6]. Furthermore, this study has shown how outpatient care significantly reduces the cost per patient, resulting in savings equivalent to the cost of a hospital stay of between 2 and 4 days per patient. This represents a significant reduction in healthcare expenses, which in turn has a positive impact on the National Health System.

In summary, both individual predictive models, one for bleeding risk and another hospital stay duration, are promising tools that must be used with caution. Their predictive power should be validated and refined over time as the dataset grows and more events are recorded. With this approach, patients can be appropriately selected for outpatient TOMES cordectomies based on their individual profiles, contributing to reduced surgical wait times, fewer hospital admissions, and overall lower healthcare costs. Additionally, minimizing hospital stay may help reduce the incidence of inpatient complications and promote a more comfortable surgical experience for patients, ultimately improving their quality of life.

## 5. Conclusions

Cordectomies performed via transoral microelectrode surgery (TOMES) are common procedures routinely carried out in hospital settings. Type III, IV, and V cordectomies performed using this technique have demonstrated a low rate of immediate postoperative bleeding complications. In this series, the latest bleeding event occurred 3.5 h after surgery, suggesting that selected cases can be safely managed in an outpatient setting with an appropriately postoperative monitoring. Moreover, extended cordectomies have been associated with a higher risk of bleeding.

When considering this approach, it is essential to make individualized decisions using a personalized predictive model that incorporates patient-specific risk factors, comorbidities, and the planned type of cordectomy. This strategy has the potential to improve patient quality of life and reduce healthcare costs.

## Figures and Tables

**Table 1:** Demographics of the sample (SD: standard deviation; Min: minimum; Max: maximum; VCP: vocal cord paralysis; SCC: squamous cell carcinoma; VeC: verrucous carcinoma).

	Total
Total	143 (100%)
Sex	
Men	131 (91.6%)
Women	12 (8.4%)
Age	
Mean (SD)	65.1 (12.5)
Median (Min.–Max.)	66 (31–94)
<60 years	37 (25.9%)
60–69 years	50 (35.0%)
≥70 years	56 (39.2%)
Disease	
VCP	16 (11.2%)
SCC	122 (85.3%)
VeC	5 (3.5%)
Toxic habits	
No	35 (24.5%)
Yes	108 (75.5%)
Tobacco	84 (58.7%)
Alcohol	71 (49.7%)

**Table 2:** Summary of variables included in univariate and multivariate predictive model with an 95% confidence interval (CI) (SCC-VeC: squamous cell carcinoma–verrucous carcinoma; VCP: vocal cord paralysis).

Variable	Bleeding	No Bleeding	Univariate Model *p*-Value	Multivariate Model *p*-Value
Total-143 (100%)	5 (3.5%)	138 (96.5%)		
Type of cordectomy			0.017	0.005
Type III—transmuscular	0	76(100%)		
Type IV/V–total/extended	5 (7.5%)	62 (92.5%)		
Anticoagulant therapy			0.773	
No	5 (3.7%)	131 (96.3%)		
Yes	0 (0.0%)	7 (100%)		
Disease			0.357	
SCC-VeC	123 (96.85%)	4 (3.15%)		
VCP	1 (6.3%)	15 (93.8%)		
Sex			0.234	0.056
Men	4 (3.1%)	127 (96.9%)		
Women	1 (8.3%)	11 (91.7%)		
Age			0.571	
<60 years	1 (2.7%)	36 (97.3%)		
60–69 years	4 (8.0%)	46 (92.0%)		
>70 years	0 (0.0%)	56 (100.0%)		
Smokers			0.391	
Yes	4 (4.8%)	80 (93.6%)		
No	1 (1.7%)	58 (98.3%)		
Alcoholism			0.663	
Yes	2 (2.8%)	68 (95.8%)		
No	3 (4.2%)	70 (97.2%)		
Chronic bronchitis			0.127	0.136
Yes	1 (12.5%)	7 (87.5%)		
No	4 (30.0%)	131 (97.0%)		

## Data Availability

Data is contained within the article.

## References

[1] SEER Cancer of the Larynx—Cancer Stat Facts. https://seer.cancer.gov/statfacts/html/laryn.html.

[2] Feng Y., Wang B., Wen S. (2011). Laser surgery versus radiotherapy for T1–T2N0 glottic cancer: A meta-analysis. ORL J. Otorhino-Laryngol. Relat. Spec..

[3] Vaculik M.F., MacKay C.A., Taylor S.M., Trites J.R.B., Hart R.D., Rigby M.H. (2019). Systematic review and meta-analysis of T1 glottic cancer outcomes comparing CO_2_ transoral laser microsurgery and radiotherapy. J. Otolaryngol.-Head Neck Surg..

[4] Baird B.J., Sung C.K., Beadle B.M., Divi V. (2018). Treatment of early-stage laryngeal cancer: A comparison of treatment options. Oral Oncol..

[5] Diaz-De-Cerio P., Preciado J., Santaolalla F., Sanchez-Del-Rey A. (2013). Cost-minimisation and cost-effectiveness analysis comparing transoral CO_2_ laser cordectomy, laryngofissure cordectomy and radiotherapy for the treatment of T1–2, N0, M0 glottic carcinoma. Eur. Arch. Oto-Rhino-Laryngol..

[6] Basterra J., Zapater E., Moreno R., Hernández R. (2006). Electrosurgical endoscopic cordectomy with microdissection electrodes: A comparative study with CO_2_ laser. J. Laryngol. Otol..

[7] Weingarten T.N., Bojanić K., Scavonetto F., Sprung J. (2013). Management of delayed hemorrhage after partial vocal cord cordectomy. J. Clin. Anesth..

[8] Remacle M., Eckel H.E., Antonelli A., Brasnu D., Chevalier D., Friedrich G., Olofsson J., Rudert H.H., Thumfart W., de Vincentiis M. (2000). Endoscopic cordectomy: A proposal for a classification by the working committee, European Laryngological Society. Eur. Arch. Oto-Rhino-Laryngol..

[9] Remacle M., Van Haverbeke C., Eckel H., Bradley P., Chevalier D., Djukic V., de Vicentiis M., Friedrich G., Olofsson J., Peretti G. (2007). Proposal for revision of the European Laryngological Society classification of endoscopic cordectomies. Eur. Arch. Oto-Rhino-Laryngol..

[10] Poves-Álvarez R., Gómez-Sánchez E., Martínez-Rafael B., Bartolomé C., Alvarez-Fuente E., Muñoz-Moreno M.F., Eiros J.M., Tamayo E., Gómez-Pesquera E. (2021). Parental satisfaction with autonomous pediatric ambulatory surgery units. Qual. Manag. Health Care.

[11] Nordin A.B., Shah S.R., Kenney B.D. (2018). Ambulatory pediatric surgery. Semin. Pediatr. Surg..

[12] Dor A., Luo Q., Gerstein M.T., Malveaux F., Mitchell H., Markus A.R. (2018). Cost-effectiveness of an Evidence-Based Childhood Asthma Intervention in Real-World Primary Care Settings. J. Ambul. Care Manag..

[13] Vogel B., Mehran R. (2016). Arterial hypertension: A neglected risk for bleeding. Catheter. Cardiovasc. Interv..

[14] Proietti M., Romiti G.F., Romanazzi I., Farcomeni A., Staerk L., Nielsen P.B., Lip G.Y. (2018). Restarting oral anticoagulant therapy after major bleeding in atrial fibrillation: A systematic review and meta-analysis. Int. J. Cardiol..

[15] Olszewski E. (1976). Blood Vascular System in Cancer of the Larynx. Arch. Otolaryngol..

[16] Altuna X., Zulueta A., Algaba J. (2005). CO_2_ laser cordectomy as a day-case procedure. J. Laryngol. Otol..

[17] Hans S., Baudouin R., Circiu M.P., Couineau F., Lisan Q., Crevier-Buchman L., Lechien J.R. (2022). Laryngeal Cancer Surgery: History and Current Indications of Transoral Laser Microsurgery and Transoral Robotic Surgery. J. Clin. Med..

